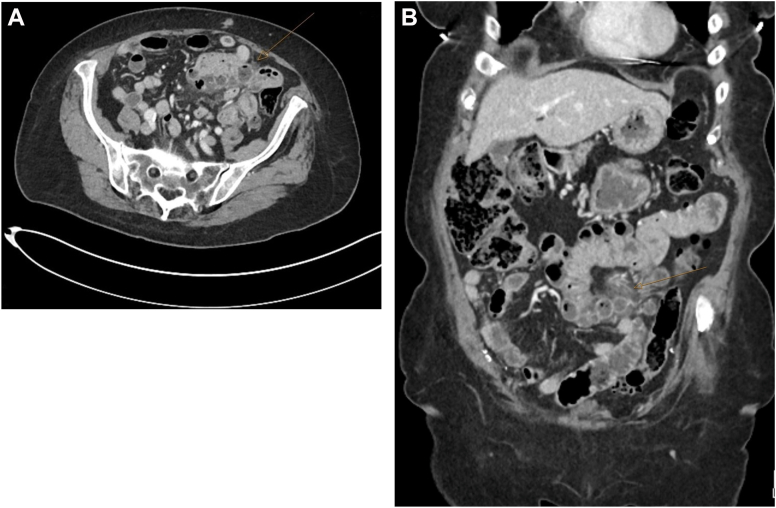# Recurrent Jejunal Diverticulitis: A Rare Cause of Acute Abdomen

**DOI:** 10.1016/j.gastha.2025.100849

**Published:** 2025-11-22

**Authors:** Osamah Al-obaidi, Sheila Cheng, Emily Nash

**Affiliations:** 1 Macquarie University Hospital, Macquarie Park, New South Wales, Australia; 2 Department of Radiology, Royal Prince Alfred Hospital, Camperdown, New South Wales, Australia; 3 AW Morrow Gastroenterology and Liver Centre, Royal Prince Alfred Hospital, Camperdown, New South Wales, Australia; 4 School of Medicine, The University of Sydney, Camperdown, New South Wales, Australia

A 67-year-old female presented with 2 days of severe generalized abdominal pain, nausea, and anorexia. She had a similar episode 6 years earlier, diagnosed as acute jejunal diverticulitis and managed conservatively. On examination, her temperature was 37.8 °C, and her abdomen was mildly distended with periumbilical tenderness. Laboratory tests revealed elevated white cell count (15.9 x 10ˆ9/L) and C-reactive protein (98 mg/L), with normal serum lactate. Contrast-enhanced computed tomography of the abdomen showed a long segment of jejunal mural thickening with surrounding fat stranding, suggesting jejunal enteritis. On further review, multiple jejunal diverticula were observed in these areas, compatible with acute jejunal diverticulitis (Figure A and Figure B). She was treated with intravenous fluid and antibiotics, bowel rest, and analgesia. Over several days, her abdominal pain gradually improved, and inflammatory markers down trended. Serial abdominal examinations showed no clinical deterioration. She was transitioned to oral antibiotics and discharged home on day six. At the 3-month follow-up, she was well, with no recurrence of symptoms. Jejunal diverticulitis is rare but potentially serious and can mimic other conditions, highlighting the need for prompt diagnosis and treatment.

**Figure:**